# Social Network Sensors for Early Detection of Contagious Outbreaks

**DOI:** 10.1371/journal.pone.0012948

**Published:** 2010-09-15

**Authors:** Nicholas A. Christakis, James H. Fowler

**Affiliations:** 1 Faculty of Arts & Sciences, Harvard University, Boston, Massachusetts, United States of America; 2 Health Care Policy Department, Harvard Medical School, Boston, Massachusetts, United States of America; 3 School of Medicine, University of California San Diego, La Jolla, California, United States of America; 4 Division of Social Sciences, University of California San Diego, La Jolla, California, United States of America; Indiana University, United States of America

## Abstract

Current methods for the detection of contagious outbreaks give contemporaneous information about the course of an epidemic at best. It is known that individuals near the center of a social network are likely to be infected sooner during the course of an outbreak, on average, than those at the periphery. Unfortunately, mapping a whole network to identify central individuals who might be monitored for infection is typically very difficult. We propose an alternative strategy that does not require ascertainment of global network structure, namely, simply monitoring the friends of randomly selected individuals. Such individuals are known to be more central. To evaluate whether such a friend group could indeed provide early detection, we studied a flu outbreak at Harvard College in late 2009. We followed 744 students who were either members of a group of randomly chosen individuals or a group of their friends. Based on clinical diagnoses, the progression of the epidemic in the friend group occurred 13.9 days (95% C.I. 9.9–16.6) in advance of the randomly chosen group (i.e., the population as a whole). The friend group also showed a significant lead time (*p*<0.05) on day 16 of the epidemic, a full 46 days before the peak in daily incidence in the population as a whole. This sensor method could provide significant additional time to react to epidemics in small or large populations under surveillance. The amount of lead time will depend on features of the outbreak and the network at hand. The method could in principle be generalized to other biological, psychological, informational, or behavioral contagions that spread in networks.

## Introduction

Current methods for the detection of contagious outbreaks ideally give contemporaneous information about the course of an epidemic, though, more typically, the indicators lag behind the epidemic.[1]–[3] However, the situation could be improved, possibly significantly, if detection methods took advantage of a potentially informative property of social networks: during a contagious outbreak, individuals at the center of a network are likely to be infected sooner than random members of the population. Hence, the careful collection of information from a sample of central individuals within human social networks could be used to detect contagious outbreaks *before* they happen in the population-at-large.

**Figure 1:**
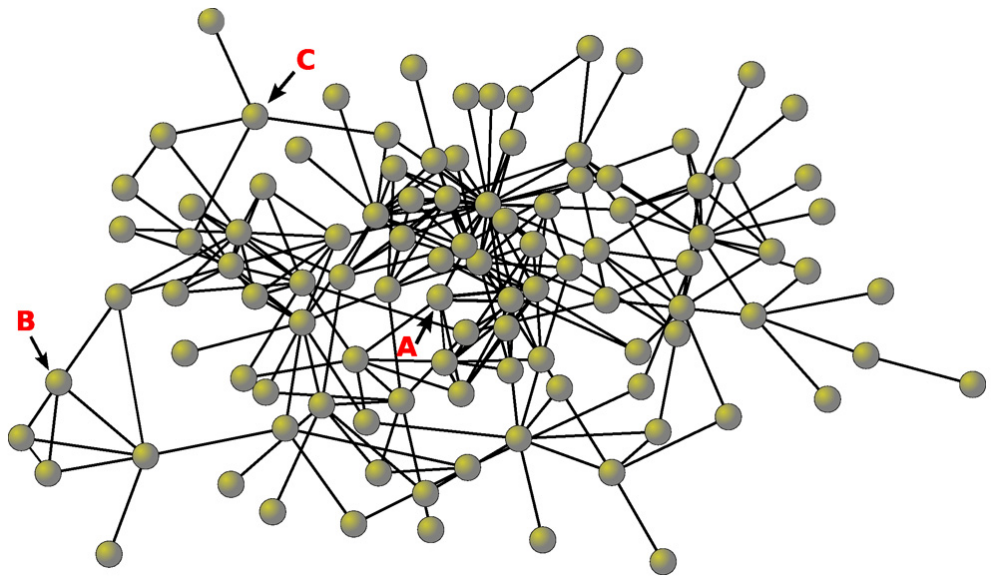
Network Illustrating Structural Parameters. This real network of 105 students shows variation in structural attributes and topological position. Each circle represents a person and each line represents a friendship tie. Nodes A and B have different “degree,” a measure that indicates the number of ties. Nodes with higher degree also tend to exhibit higher “centrality” (node A with six friends is more central than B and C who both only have four friends). If contagions infect people at random at the beginning of an epidemic, central individuals are likely to be infected sooner because they lie a shorter number of steps (on average) from all other individuals in the network. Finally, although nodes B and C have the same degree, they differ in “transitivity” (the probability that any two of one's friends are friends with each other). Node B exhibits high transitivity with many friends that know one another. In contrast, node C's friends are not connected to one another and therefore they offer more independent possibilities for becoming infected earlier in the epidemic.

**Figure 2:**
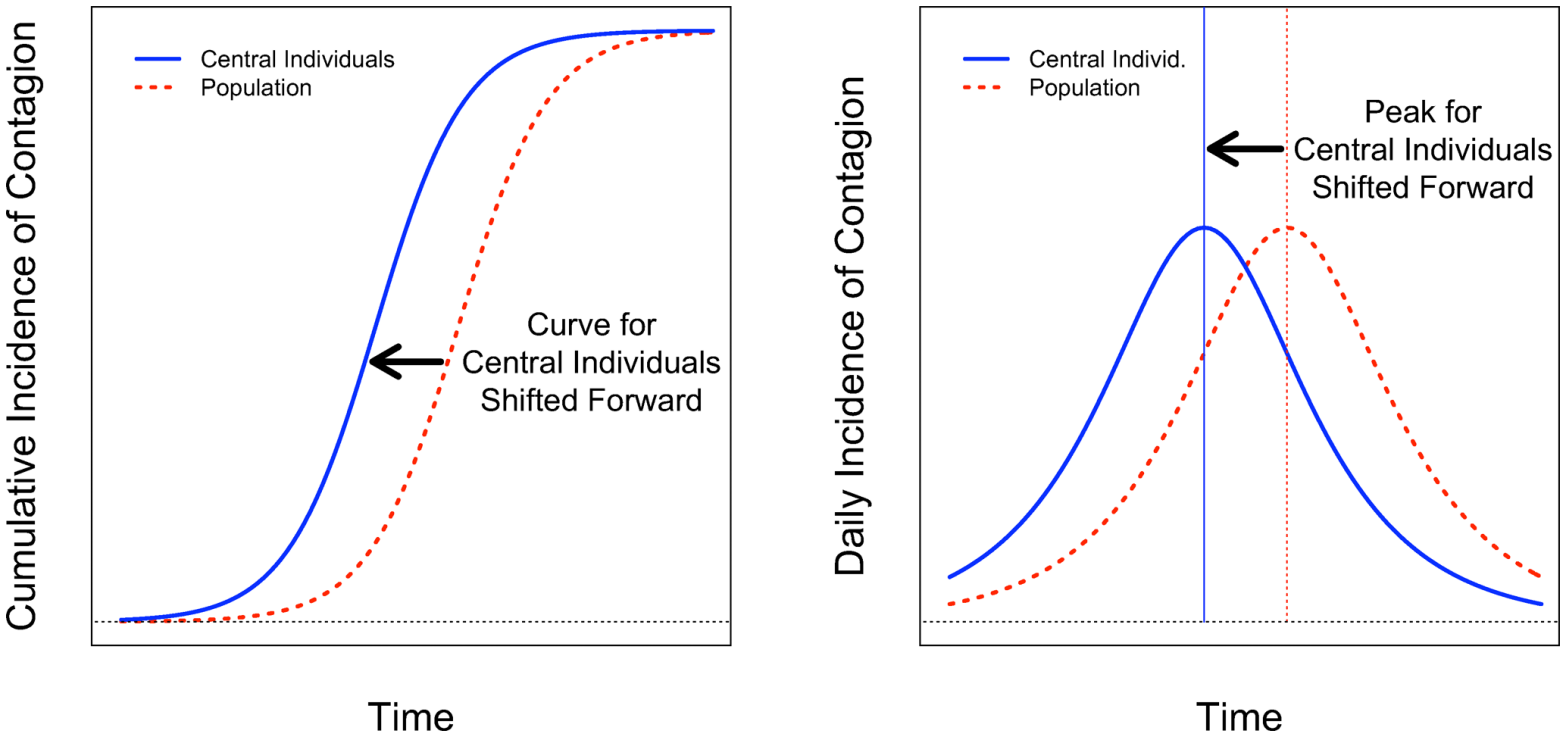
Theoretical expectations of differences in contagion between central individuals and the population as a whole. A contagious process passes through two phases, one in which the number of infected individuals exponentially increases as the contagion spreads, and one in which incidence exponentially decreases as susceptible individuals become increasingly scarce. These dynamics can be modeled by a logistic function. Central individuals lie on more paths in a network compared to the average person in a population and are therefore more likely to be infected early by a contagion that randomly infects some individuals and then spreads from person to person within the network. This shifts the S-shaped logistic cumulative incidence function forward in time for central individuals compared to the population as a whole (left panel). It also shifts the peak infection rate forward (right panel).

A contagion that stochastically infects some individuals and then spreads from person to person in the network will tend, on average, to reach centrally located individuals more quickly than peripheral individuals because central individuals (as defined in various ways described below) are a smaller number of steps (degrees of separation) away from the average individual in the network (see Figure 1).[4]–[6] Indeed, although some contagions can spread via incidental contact, the duration of exposure between people with social ties is typically much higher than between strangers, suggesting that the social network itself will be an important conduit for the spread of an outbreak.[5], [7] As a result, we would expect the S-shaped epidemic curve [8], [9] to be shifted to the left (forward in time) for centrally located individuals compared to the population as a whole (see Figure 2). This shift, if it could be observed, would allow for early detection of an outbreak.

Prior modeling research suggests that vaccinating central individuals in networks could enhance the population-level efficacy of a prophylactic intervention [10]–[13] and other work suggests that optimal placement of sensors in physical networks (such as water pumping stations) could detect outbreaks sooner.[14] However, mapping a whole network to identify particular individuals from whom to collect information is costly, time-consuming, and often impossible, especially for large networks.

We therefore explore a novel, alternative strategy that does *not* require ascertainment of global network structure, namely, *monitoring the friends of randomly selected individuals*. This strategy exploits an interesting property of human social networks: on average, the friends of randomly selected people possess more links (have higher degree) and are also more central (e.g., as measured by betweenness centrality) to the network than the initial, randomly selected people who named them.[15]–[19] Therefore, we expect a set of nominated friends to get infected earlier than a set of randomly chosen individuals (who represent the population as a whole). More specifically, a random sample of individuals from a social network will have a mean degree of *μ* (the mean degree for the population); but the friends of these random individuals will have a mean degree of *μ* plus a quantity defined by the variance of the degree distribution divided by *μ*. Hence, when there is variance in degree in a population, and especially when there is high variance, the mean number of contacts for the friends will be greater (and potentially much greater) than the mean for the random sample. This is sometimes known as the “friendship paradox” (“your friends have more friends than you do”) [15]–[19].

While the idea of *immunizing* such friends of randomly chosen people has previously been explored in a stimulating theoretical paper [12], to our knowledge, a method that uses nominated friends as *sensors* for early detection of an outbreak has not previously been proposed, nor has it been tested on any sort of real outbreak. To evaluate the effectiveness of nominated friends as social network sensors, we therefore monitored the spread of flu at Harvard College from September 1 to December 31, 2009. In the fall of 2009, both seasonal flu (which typically kills 41,000 Americans each year [20]) and the H1N1 strain were prevalent in the US, though the great majority of cases in 2009 have been attributed to the latter.[1] It is estimated that this H1N1 epidemic, which began roughly in April 2009, infected over 50 million Americans. Unlike seasonal flu, which typically affects individuals older than 65, H1N1 tends to affect young people. Nationally, according to the CDC, the epidemic peaked in late October 2009, and vaccination only became widely available in December 2009. Whether another outbreak of H1N1 will occur (for example, in areas and populations that have heretofore been spared) is a matter of some debate,[1] but many scholars have been studying the situation from biological and public health perspectives.[21], [22]

We enrolled a total of 744 undergraduate students from Harvard College, discerned their friendship ties, and tracked whether they had the flu beginning on September 1, 2009 (from the start of the new academic year) to December 31, 2009. This sample was assembled by empanelling two groups of students of essential analytic interest: (1) a sample chosen randomly from the 6,650 Harvard undergraduates (N = 319), and (2) a “friends” sample (N = 425) composed of individuals who were named as a friend at least once by a member of this random sample (see Supporting Information Text S1 for more details).

In addition, as a byproduct of empanelling the foregoing group of 744 students, we wound up having information about a total of 1,789 uniquely identified Harvard College students (who either participated in the study or who were nominated as friends or as friends of friends); we used this information to draw the social network of part of the Harvard College student body (see Supporting Information Text S1 for more details).

All subjects completed a brief background questionnaire soliciting demographic information, flu and vaccination status since September 1, 2009, and certain self-reported measures of popularity. We also obtained basic administrative data from the Harvard College registrar, such as sex, class of enrolment, and inter-collegiate sports participation.

We tracked cases of formally diagnosed influenza among the students in our sample as recorded by University Health Services (UHS) beginning on September 1, 2009 through December 31, 2009. Presenting to the health service indicates a more severe level of symptomatology, of course, and so we do not expect the same overall prevalence using this diagnostic standard as with self-reported flu discussed below. However, UHS data offer the advantage of allowing us to obtain information about flu symptoms as assessed by medical staff.

Beginning on October 23, 2009, we also collected self-reported flu symptom information from participants via email twice weekly (on Mondays and Thursdays), continuing until December 31, 2009. The students were queried about whether they had had a fever or flu symptoms since the last email contact, and there was very little missing data (47% of the subjects completed *all* of the biweekly surveys, and 90% missed no more than two of the surveys).

Self-report of symptoms rather than serological testing is the current standard for flu diagnosis. Similar to previous studies,[23] students were deemed to have a case of flu (whether seasonal or the H1N1 variety) if they reported having a fever of greater than 100° F (37.8°C) *and* at least two of the following symptoms: sore throat; cough; stuffy or runny nose; body aches; headache; chills; or fatigue. We checked the sensitivity of our findings by using definitions of flu that required more symptoms, and our results did not change (see Supporting Information Text S1). As part of the foregoing biweekly self-reports, in order to complement the UHS vaccination records, we also ascertained whether the students reported having been vaccinated (with seasonal flu vaccine or H1N1 vaccine or both) at places other than (and including) UHS.

To be clear, we are not suggesting that a person's precise position in the observed network, nor indeed whether he was nominated as a friend or not (and by whom), traces out the *actual path* by which he acquired (or did not acquire) the flu. The topological parameters we measured here, or indeed the fact that a person was deemed to be a member of the friend group, serve as *proxies* for the subject's actual location within what is an essentially unobservable social network (including real friends, relatives, casual contacts, and so on) through which the flu spreads by inter-personal means. Being a “friend” is a marker for a person's social-network position, whatever the path of infection to this person actually is. Of course, it is likely that measured friendship networks are related to contact networks more generally: for instance, people with more friends should come into greater contact with more strangers both directly and indirectly via their friends.

## Results

By December 31, 2009, the cumulative incidence of flu in our sample was 8% based on diagnoses by medical staff, and it was 32% based on self-reports, which mirrored other studies of school-based outbreaks and also contemporaneous national estimates for the college-student population.[23], [24] As expected, the prevalence was higher by the self-report standard. We studied the association of several demographic and other variables with cumulative flu incidence at day 122 (the last day of follow-up) to see whether they predicted an increase in overall risk. None of these variables was significantly associated with flu diagnoses by medical staff (see Supporting Information Text S1), so we focused on the effect of these variables on *shifts in the timing* of the distribution.

**Figure 3:**
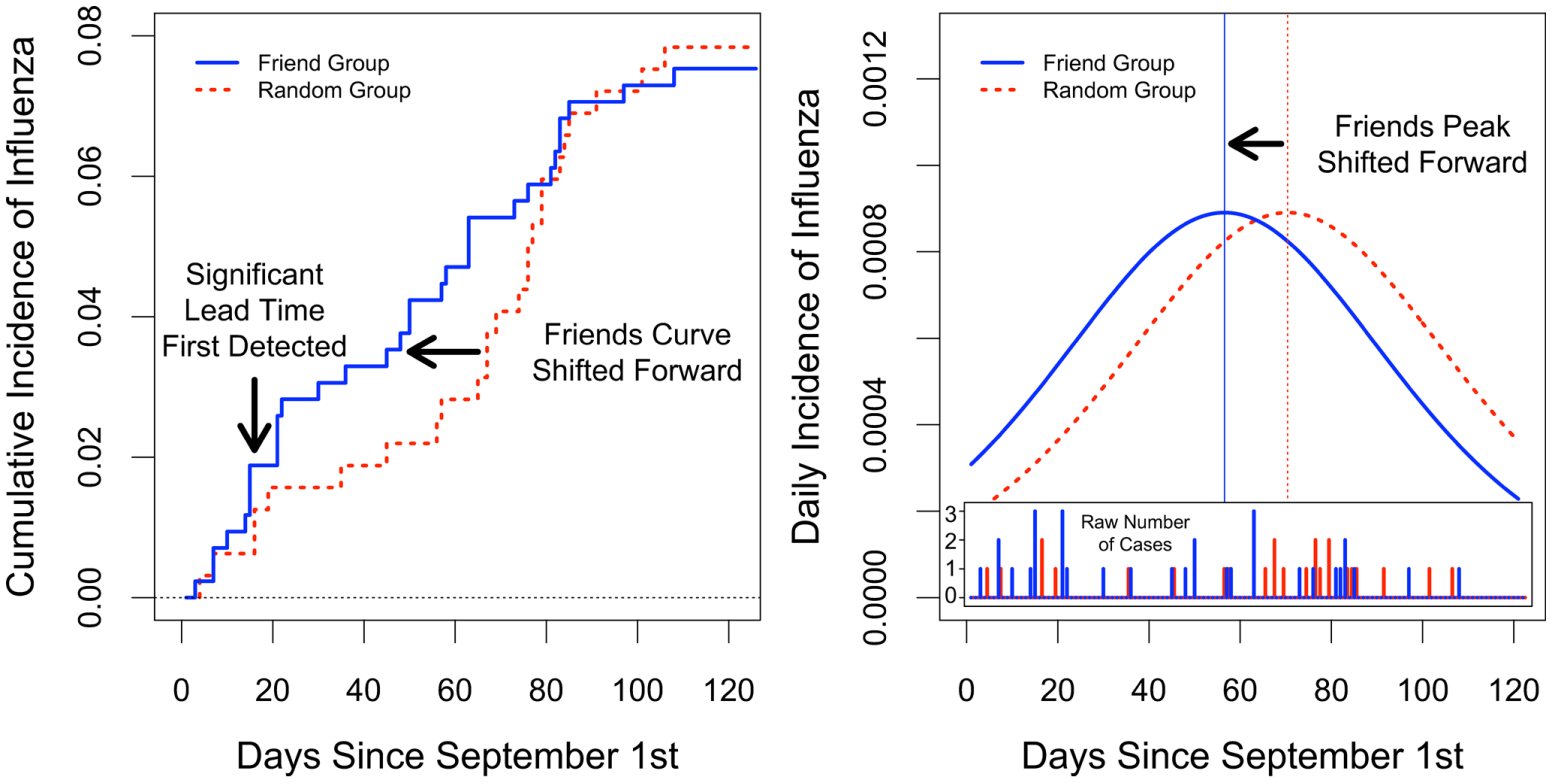
Empirical differences in flu contagion between “friend” group and randomly chosen individuals. We compared two groups, one composed of individuals randomly selected from our population, and one composed of individuals who were nominated as a friend by members of the random group. The friend group was observed to have significantly higher measured in-degree and betweenness centrality than the random group (see Supporting Information Text S1). In the left panel, a nonparametric maximum likelihood estimate (NPMLE) of cumulative flu incidence (based on diagnoses by medical staff) shows that individuals in the friend group tended to get the flu earlier than individuals in the random group. Moreover, predicted daily incidence from a nonlinear least squares fit of the data to a logistic distribution function suggests that the peak incidence of flu is shifted forward in time for the friends group by 13.9 days (right panel). A significant (*p*<0.05) lead time for the friend group was first detected with data available up to Day 16. Raw data for daily flu cases in the friend group (blue) and random group (red) is shown in the inset box (right panel).

As hypothesized, the cumulative incidence curves for the friend group and the random group diverge and then converge (Figure 3). NLS estimates suggest that the friends curve for flu diagnosed by medical staff is shifted 13.9 days forward in time (95% C.I. 9.9–16.6), thus providing early detection. This represents approximately 60% of one standard deviation in the time-to-event in the whole sample. The results also indicate a significant but smaller shift in self-reported flu symptoms (3.2 days, 95% C.I. 2.2–4.3). In the case of both the clinical and self-reported diagnostic standards, the estimates are robust to a number of control variables including H1N1 vaccination, seasonal flu vaccination, sex, college class, and inter-collegiate sports participation (see Supporting Information Text S1).

The foregoing estimates rely on full information *ex post*, but we wondered when it would also be possible to detect a difference in the friend group and the random group in real time, given less complete data. We therefore estimated the models each day using all available information up to that day. For flu diagnoses by medical staff, the friend group showed a significant lead time (*p*<0.05) on day 16, a full 46 days before the estimated peak in daily incidence in visits to the health service. For self-reported flu symptoms, the friend group showed a significant lead time by day 39, which is 83 days prior to the estimated peak in daily incidence in self-reported symptoms. Thus, a comparison of outcomes in the friends group and the randomly chosen group could be an effective technique for detecting outbreaks at early stages of an epidemic.

A possible alternative to the friendship nomination procedure would be to rely on self-reported popularity or self-reported counts of numbers of friends in order to identify a high-risk group. We measured our subjects' self-perceptions of popularity using an eight-item scale, but this did not yield a significant shift forward in time for flu diagnoses (see Supporting Information Text S1). Moreover, controlling for self-reported popularity did not alter the significance of the lead time provided by the friend group for either flu diagnoses by medical staff or self-reported flu symptoms. These results suggest that being nominated as a friend captures more network information (including the tendency to be central in the network) than self-reported network attributes. Such information collected about one person, from another, might also be more accurate [12].

**Figure 4:**
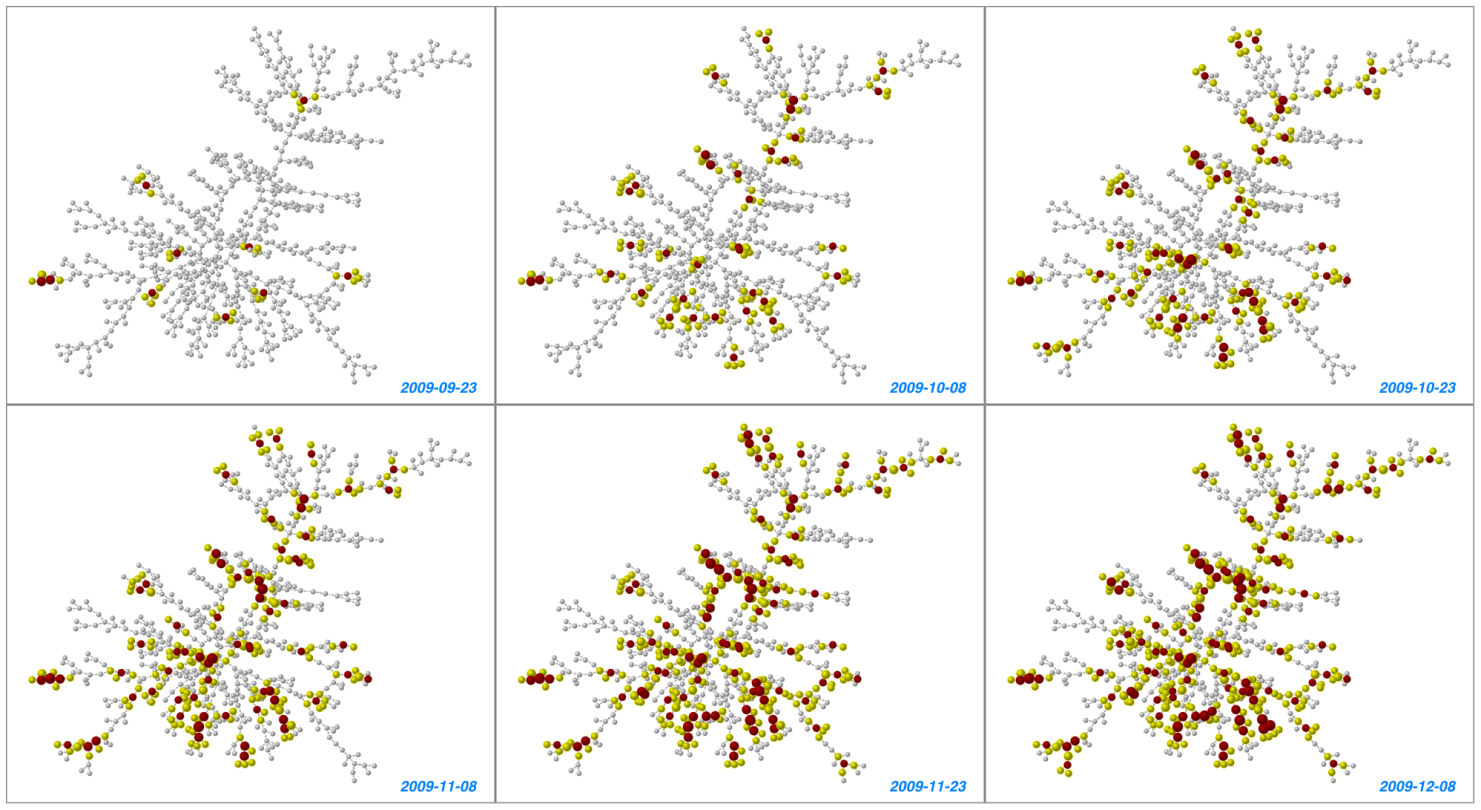
Progression of flu contagion in the friendship network over time. Each frame shows the largest component of the network (714 people) for a specific date, with each line representing a friendship nomination and each node representing a person. Infected individuals are colored red, friends of infected individuals are colored yellow, and node size is proportional to the number of friends infected. All available information regarding infections is used here. Frames for all 122 days of the study are available in a movie of the epidemic posted in the Supporting Information (Video S1).

Although the method described here does *not* require information about the full network, our survey took place on a college campus in which many nominators were themselves nominated, and the same person was frequently nominated several times. Hence, our data collection procedures wound up yielding information about 1,789 unique, inter-connected students who were either surveyed or were identified as friends by those who took part in the study. A connected component of 714 people was in turn apparent within these 1,789 individuals. We illustrate the spread of flu in this component in Figure 4, which shows the tendency of the flu to “bloom” in more central nodes of the network, and also in a 122-frame movie of daily flu prevalence available online (see Supporting Information Video S1).

Sampling a densely interconnected population also allowed us to actually measure egocentric network properties like in-degree (number of times a subject was nominated as a friend), betweenness centrality (the number of shortest paths in the network that pass through an individual), coreness (the number of friends an individual has when all individuals with fewer friends are iteratively removed from the network), and transitivity (the probability that two of one's friends are friends with one another). This would not be possible in a deployment of the friends' technique in larger populations (wherein surveyed individuals would be much less likely to actually be connected to each other). The results showed that, as expected, the friend group differed significantly from the random group for all these measures, exhibiting higher in-degree (Mann Whitney U test *p*<0.001), higher centrality (*p<*0.001), higher *k*-coreness (*p<*0.001), and lower transitivity (*p = *0.039).

**Figure 5:**
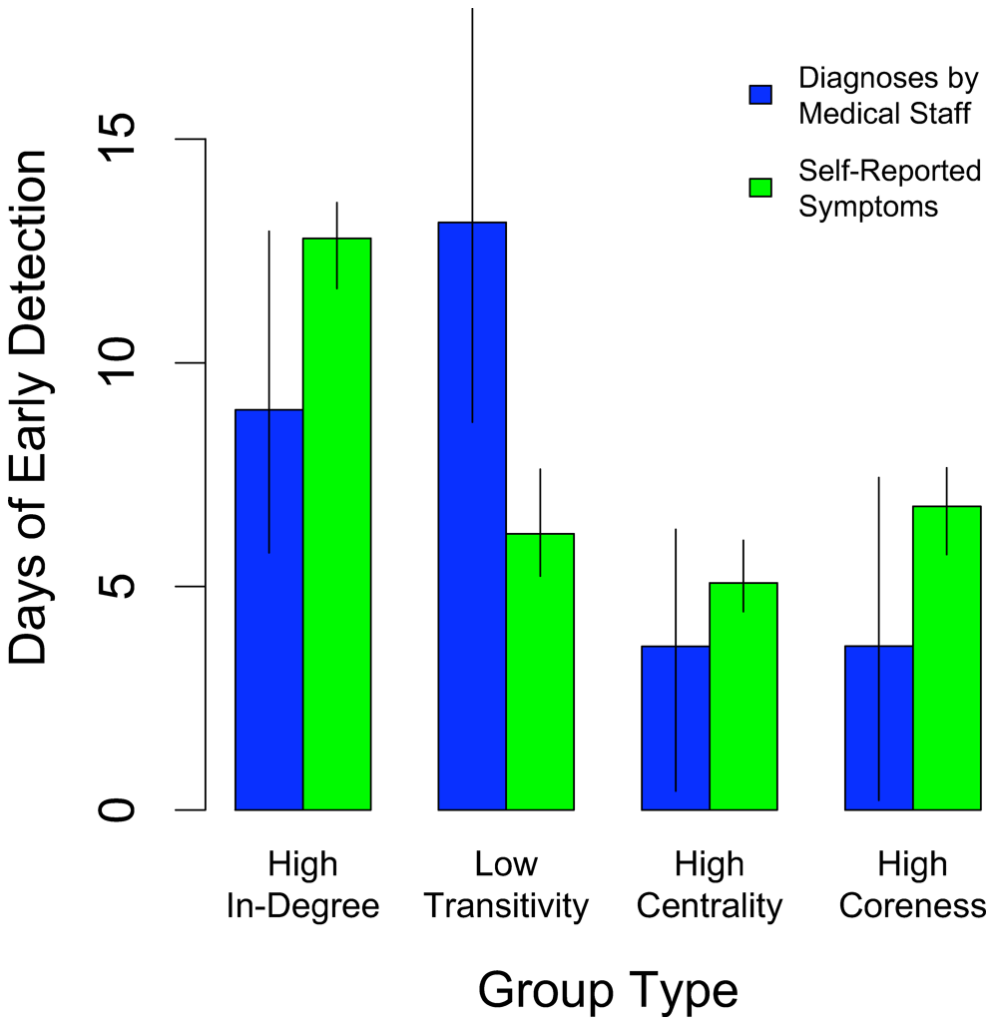
Estimated days of advance detection of a flu outbreak when following specific groups. Here, degree, transitivity, centrality, and coreness are computed based on the mapping of the network. The high in-degree group is composed of individuals who have a higher-than-average number of other people in the network who name them as a friend. The low transitivity group is composed of individuals with below-average probability that any two of their friends are friends with one another. The high centrality group is composed of individuals with a higher-than-average betweenness, which is the number of shortest paths connecting all individuals in a network that pass through a given person. The high coreness group is composed of individuals with a higher-than-average coreness, which is the number of friends a person has once all individuals with fewer friends have been eliminated from the network. Analyses were conducted separately for data based on flu diagnoses by medical staff (blue bars) and data based on self-reported flu symptoms (green bars). Estimates and 95% confidence intervals are based on a nonlinear least squares fit of the flu data to a logistic distribution function (see Supporting Information Text S1). The results show that flu outbreaks occur up to two weeks earlier in each of these groups.

We hypothesized that each of these measures could help to identify groups that could be used as social network sensors when full network information is, indeed, available (see Figure 5). For example, we expect in-degree to be associated with early contagion because more friends means more paths to others in the network who might be infected. NLS estimates suggest that each additional nomination shifts the flu curve left by 5.7 days (95% C.I. 3.6–8.1) for flu diagnoses by medical staff and 8.0 days (95% C.I. 7.3–8.5) for self-reported symptoms. On the other hand, the same is not true for out-degree (the number of friends a person names). Pertinently, this is the only quantity that would be straightforwardly ascertainable by asking respondents about themselves. However, there is low variance in this measure in the present setting since most people named three friends (the maximum allowed by our survey).

We also expect betweenness centrality to be associated with early contagion. NLS estimates suggest that individuals with maximum observed centrality shift the flu curve left by 16.5 days (95% C.I. 1.9–28.3) for flu diagnoses by medical staff and 22.9 days (95% C.I. 20.0–27.2) for self-reported symptoms, relative to those with minimum centrality. A related measure, *k-*coreness, also suggests that people at the center of the network get the flu earlier. NLS estimates suggest that increasing the measure *k* by one (the range is from 0 to 3) shifts the flu curve left by 4.3 days (95% C.I. 1.8–6.5) for flu diagnoses by medical staff and 7.5 days (95% C.I. 6.8–8.2) for self-reported symptoms. Moreover, both betweenness centrality and *k*-coreness remain significant even when controlling for both in-degree and out-degree, suggesting that it is not just the number of friends that is important with respect to flu risk, but also the number of friends of friends, friends of friends of friends, and so on [6].

Finally, we expect transitivity to be negatively associated with early contagion. People with high transitivity may be poorly connected to the rest of the network because their friends tend to know one another and exist in a tightly knit group. In contrast, those with low transitivity tend to be connected to many different, independent groups, and each additional group increases the possibility that someone in that group has the flu and that it spreads to the subject. NLS estimates suggest that individuals with minimum observed transitivity shift the flu curve left by 31.9 days (95% C.I. 23.5–43.5) for flu diagnoses by medical staff and 15.0 days (95% C.I. 12.7–18.5) for self-reported symptoms compared to those with maximum transitivity. Moreover, transitivity remains significant even when controlling for both in-degree and out-degree.

## Discussion

For many contagious diseases, early knowledge of when – or whether – an epidemic is unfolding is crucial to policy makers and public health officials responsible for defined populations, whether small or large. In fact, with respect to flu, models assessing the impact of prophylactic vaccination in a metropolis such as New York City suggest that vaccinating even one third of the population would save lives and shorten the course of the epidemic, but only if implemented a month earlier than usual.[25], [26] A method like the one described here could help provide just such early detection.

In fact, this method could be used to monitor targeted populations of any size, in real time. For example, a health service at a university (or other institution) could empanel a sample of subjects who are nominated as friends and who agree to be passively monitored for their health care use (e.g., in the form of visits to health care facilities); a spike in cases in this group could be read as a warning of an impending outbreak. Public health officials responsible for a city could also empanel a sample of randomly chosen individuals and a sample of nominated friends (perhaps a thousand people in all) who have agreed to report their symptoms using brief, periodic text messages or an online survey system (like the one employed here). Regional or national populations could also be monitored in this fashion, with a sample of nominated friends being periodically surveyed instead of, or in addition to, a random sample of people (as is usually the norm). Since public health officials often monitor populations in any case, the change in practice required to monitor a sample of these more central individuals might not be too burdensome.

Moreover, whereas officials responsible for a single, relatively small institution might possibly actively seek out central individuals to vaccinate them (hence potentially confounding the utility of such individuals as sensors), such a focused vaccination effort would be unlikely to be initiated with a regional or national sample, given the likely irrelevance of vaccinating the actual sensor sample members as a means to control any wide-scale epidemic. Regardless, since the people being followed as sensors would, in most cases, be only a small fraction of all the central people in a population (let alone of all the people in the population as a whole), even if they were actually treated (after an epidemic were noted to have affected them), it seems unlikely that this would materially affect the course of the epidemic or compromise the utility of the central individuals as sensors. Nevertheless, mathematical modelling of such procedures would help us to better understand what role sensors might play in helping to reverse the course of an epidemic.

The difference in the timing of the course of the epidemic in the friend and random groups could be exploited in at least two different ways. First, if solely the friends group were being monitored, an analyst tracking an outbreak might look for the first evidence that the incidence of the pathogen among the friends group rose above a predetermined rate (e.g., a noticeable increase above a zero background rate); this itself could indicate an impending epidemic. Second, in a strategy that would yield more information, the analyst could track both a sample of friends and a sample of random subjects, and the harbinger of an epidemic could be taken to be when the two curves were seen to first diverge from each other. Especially in the case of the spread of contagions other than biological pathogens, the difference between these two curves provides additional information: the adoption curve among the random sample provides evidence of secular trends in the population, whereas the *difference* between the two curves provides evidence of a network (inter-personal) effect, over and above the baseline force of the epidemic.

While our goal here was to evaluate how the method of surveying friends could provide early detection of contagious outbreaks in general, it is noteworthy that, in the specific case of the flu, the method we evaluated appears to provide longer lead times than other extant methods of monitoring flu epidemics. Current surveillance methods for the flu, such as those implemented by the CDC that require collection of data from subjects seeking outpatient care or having lab tests, are typically lagging indicators about the timing of the epidemic (information is typically one to two weeks behind the actual course of the epidemic).[1] A proposal to use Google Trends to monitor online searches for information about the flu suggests that this approach could offer a better indicator, providing evidence of an outbreak at least a week before published CDC reports.[2], [3] Another innovative proposal involved the use of a prediction market that also accelerated the warning [27]. However, while potentially instantaneous, the Google Trends and prediction market methods would only, at best, give *contemporaneous* information about rates of infection. In contrast, we show that the sensor method described here can detect an outbreak of flu two weeks *in advance*. That is, the sensor network method provides *early detection* rather than just *rapid warning*.

Moreover, the sensor method could be used in conjunction with online search. By following the online behavior of a friend group, or a group known to be central in a network (for example, based on e-mail records which could be used to reconstruct social network topology), Google or other search engines might be able to get high-quality, real-time information about the epidemic with even greater lead time, giving public health officials even more time to plan a response.

How much advance detection would be achieved for other pathogens or in populations of different size or composition remains unknown. The ability of the proposed method to detect outbreaks early, and how early it might do so, will depend on intrinsic properties of the thing that is spreading (e.g., the biology of the pathogen); how this thing is measured; the nature of the population, including the overall prevalence of susceptible or affected individuals; the number of people empanelled into the sensor group; the topology of the network (for example, the degree distribution and its variance, or other structural attributes) [6], [28]; and other factors, such as whether the outbreak modifies the structure of the network as it spreads (for example, by killing people in the network, or, in the case of spreading information, perhaps by affecting the tendency of any two individuals to remain connected after the information is transmitted). The amount of time, in terms of early detection, provided could thus vary considerably, depending on attributes unique to each setting.

While the social network sensor strategy has been illustrated with a particular outbreak (flu) in a particular population (college students), it could potentially be generalized to other phenomena that spread in networks, whether biological (antibiotic-resistant germs), psychological (depression) [29], normative (altruism) [30], informational (rumors), or behavioral (smoking) [31]. Outbreaks of a wide variety of deleterious or desirable conditions could be detected before they have reached a critical threshold in populations of interest.

## Materials and Methods

We obtained written informed consent from all participants and the study was approved by and carried out under the guidelines of the Committee on the Use of Human Subjects in Research at Harvard University.

To measure self-perceived popularity, we adapted a set of 8 questions previously used to assess the popularity of co-workers.[32]

To ascertain friends, we asked: “We will ask that you provide us with the names and contact information of 2-3 [of your] friends…. Please provide the contact information for 2–3 Harvard College students who you know and who you think would like to participate in this study.”

We used friendship nominations to measure the *in-degree* (the number of times an individual is named as a friend by other individuals) and *out-degree* (the number of individuals each person names as a friend) of each subject. The in-degree is virtually unrestricted (the theoretical maximum is *N* – 1, the total number of other people in the network) but the out-degree is restricted to a maximum of 3, given the way we elicited friendship information.

We measured *betweenness centrality*, which identifies the extent to which an individual lies on potential paths for contagions passing from one individual to another through the network; this quantity summarizes how central an individual is in the network (see Figure 1).[33] Additionally, we measured *k*-*coreness*, which identifies the number of friends a person has after all individuals with fewer friends are iteratively removed from the network. Recent work suggests this measure may be more appropriate than centrality for understanding spreading processes in correlated networks [6]. We measured *transitivity* as the empirical probability that two of a subject's friends are also friends with each other, forming a triangle (see Figure 1). This measure is just the total number of triangles of ties between an individual and his or her social contacts divided by the total possible number of triangles.

We used Pajek [34] to draw two-dimensional pictures of the network, and we implemented the Kamada-Kawai algorithm, which generates a matrix of shortest network path distances from each node to all other nodes in the network and repositions nodes in an image so as to reduce the sum of the difference between the plotted distances and the network distances.[35] A movie of the spread of flu with a frame for each of the 122 days of the study is available online (see Supporting Information Text S1).

We calculated the cumulative flu incidence for both the friend group and the random group using a nonparametric maximum likelihood estimate (NPMLE) [36]. We also calculated the predicted daily incidence using an estimation procedure designed to measure the shift in the time course of a contagious outbreak associated with a given independent variable (see Supporting Information Text S1). In this procedure, we fit the observed probability of flu to a cumulative logistic function via nonlinear least squares (NLS) estimation [37]. To derive standard errors and 95% confidence intervals, we used a bootstrapping procedure in which we repeatedly re-sampled subject observations with replacement and re-estimated the fit [38]. This procedure produced somewhat wider confidence intervals than those based on asymptotic approximations, so we report only the more conservative bootstrapped estimates. Finally, we calculated how many days of early detection was possible for groups with various network attributes by multiplying the coefficient and confidence intervals in the foregoing models by the mean difference between the above-average group and the below-average group (see Supporting Information Text S1).

## Supporting Information

Video S1 Progression of flu contagion in the friendship network over time.
(11.14 MB MOV)

Text S1 Methods and Regression Output Tables.
(0.27 MB DOC)
