# Supplementary material for: Social Network Sensors for Early Detection of Contagious Outbreaks
Source: PLoS One. 2010 Sep 15;5(9):e12948. doi: 10.1371/journal.pone.0012948 (PMC2939797; doi:10.1371/journal.pone.0012948)
Supplement: Text S1 — Methods and Regression Output Tables. (0.27 MB DOC) [file pone.0012948.s002.doc]

## Supporting Information Text S1 for

Social Network Sensors for
Early Detection of Contagious Outbreaks

Nicholas A. Christakis^1,2*^, James H. Fowler^3,4^

^1^Faculty of Arts & Sciences, Harvard University, Boston, Massachusetts, USA

^2^Health Care Policy Department, Harvard Medical School, Boston, Massachusetts, USA

^3^School of Medicine, University of California, San Diego, La Jolla, California, USA
^4^Division of Social Sciences, University of California, San Diego, La Jolla, California, USA

∗ To whom correspondence should be addressed, email: christakis@hcp.med.harvard.edu, phone: (617) 432-5890.

**Subjects**

We enrolled a total of 744 undergraduate students from Harvard College, discerned their friendship ties, and tracked whether they had the flu beginning on September 1, 2009, from the start of the new academic year, to December 31, 2009.

Beginning on October 23, 2009, we approached 1,300 randomly selected Harvard College students (out of 6,650); we waited until a few weeks of the new school year had passed in order to be able to obtain current friendship information. Of these 1,300 students, 396 (30%) agreed to participate. All of these students were in turn asked to nominate up to three friends, and a total of 1,018 friends were nominated (average of 2.6 friends per nominator). This yielded 950 unique individuals to whom we sent the same invitation as the initial group. Of these, 425 (45%) agreed to participate. However, 77 of these 950 subjects were themselves members of the original, randomly selected group and hence were already participants. Thus, the sample size after the enrolment of the random group and the friend group was 744.

Nominated friends were sent the same survey as their nominators; hence, the original 425 friends also nominated 1,180 of their own friends (average of 2.8 friends per nominator), yielding 1004 further, unique individuals. Although we did *not* send surveys to these “friends of friends,” 303 (30%) were themselves already enrolled either in the friends group or in the initial randomly selected group.

Thus, in the end, we have empanelled two groups of students of essential analytic interest here: a “random” sample (N=319) and a “friends” sample (N=425) composed of individuals who were named as a friend at least once by a member of the random group. In addition, we ultimately had information about a total of 1,789 uniquely identified students (who either participated in the study or who were nominated as friends or friends of friends) with which to draw social networks of the Harvard College student body (27% of all 6,650 undergraduates). Our sample of 744 was thus embedded in this larger network of 1,789 people.

After giving informed consent, all subjects completed a brief background questionnaire soliciting demographic information, flu and vaccination status since September 1, 2009, and certain self-reported measures of popularity. We also obtained basic administrative data from the Harvard College registrar, such as sex, class of enrolment, and information about participation in varsity sports.

We also tracked cases of formally diagnosed influenza among the students in our sample as recorded by University Health Services (UHS) beginning on September 1, 2009 through December 31, 2009. Presenting to the health service indicates a more severe level of symptomatology, of course, and so we do not expect the same overall prevalence using this diagnostic standard as with self-reported flu discussed below. However, UHS data offer the advantage of allowing us to obtain information about flu symptoms as assessed by medical staff. A total of 627 of the 744 students (84%) who agreed to participate in the survey portion of our study also gave written permission for us to obtain their health records. Finally, 7 students reported being diagnosed with flu by medical staff at facilities other than UHS (in response to survey questions asked of all students), so we include these in the data as well.

Notably, we do not expect cases of flu to meaningfully alter the social networks and friendship patterns of Harvard undergraduates, let alone over a two-month period. And, we assume that the friendship network of Harvard students in our sample did not change meaningfully over the period September to December. That is, we treat the network as static over this time interval.

Beginning on October 23, 2009, we also collected *self-reported* flu symptom information from participants via email twice weekly (on Mondays and Thursdays), continuing until December 31, 2009. The enrolled students were queried about whether they had had a fever or flu symptoms since our last email contact, and there was very little missing data (47% of the subjects completed *all* of the biweekly surveys, and 90% missed no more than two of the surveys).

Self-report of symptoms rather than serological testing is the current standard for flu diagnosis. Students were deemed to have a case of flu (whether seasonal or the H1N1 variety) if they report having a fever of greater than 100˚ F (37.8˚ C) *and* at least two of the following symptoms: sore throat; cough; stuffy or runny nose; body aches; headache; chills; or fatigue. We checked the sensitivity of our findings by using definitions of flu that required more symptoms, and our results did not change. As part of the foregoing biweekly follow-up, and to supplement the UHS vaccination records, we also ascertained whether the students reported having been vaccinated (with seasonal flu vaccine or H1N1 vaccine or both) at places other than (and including) UHS.

Hence, we had two measures of flu incidence. The medical-staff standard was a formal diagnosis by a health professional and typically reflected more severe symptoms. The self-reported standard captured cases that did not come to formal medical attention. As expected, the cumulative incidence of the latter was approximately four times the former (32% versus 8%) by the time of cessation of follow-up on December 31, 2009.

**Network Measures**

We use friendship nominations to measure the *in-degree* (the number of times an individual is named as a friend by other individuals) and *out-degree* (the number of individuals each person names as a friend) of each subject. The in-degree is virtually unrestricted (the theoretical maximum is *N* – 1, the total number of other people in the network) but the out-degree is restricted to a maximum of 3 due to the name generator used.

We measure *betweenness centrality*, which identifies the extent to which an individual lies on potential paths for passing contagions from one individual to another through the network.[[1]](#endnote-2) If we let represent the number of shortest paths from subject *i* to subject *k*, and represent the number of shortest paths from subject *i* to subject *k* that pass through subject *j*, then the betweenness centrality measure *x* for subject *j* is . To ease interpretability we divided all scores by max(*x_j_*) so that all measures would lie between and including 0 and 1.

To measure *k*-coreness, we start by removing all individuals with one or fewer friends. After one iteration, some individuals may be left with only one friend, so we continue pruning the system iteratively until there is no individual left who has one or fewer friends. The removed nodes are all assigned a value of *k*=1. Next, we iteratively remove all individuals who have two or fewer friends, giving them a value of *k*=2. We continue this process, increasing the threshold each time, until all nodes are assigned a value (the maximum in our network is *k=*3).

Finally, we also measure *transitivity* as the empirical probability that two of a subject’s friends are also friends with each other, forming a triangle. This measure is just the total number of triangles of ties divided by the total possible number of triangles for each individual. This measure is undefined for individuals with less than 2 friends (23 cases out of 744), and so we treat this measure as missing in those cases.

Note that for the purpose of measuring transitivity, coreness, and betweenness centrality, we assume all directed ties are undirected, so that a tie in either direction becomes a mutual tie. For example, we consider the case where A names B, B names C, and C names A to be transitive. Likewise, if A names B, A names C, and B names C, we consider the relationships to be transitive for all three individuals.

We used Pajek[[2]](#endnote-3) to draw pictures of the networks and used the Kamada-Kawai algorithm, which generates a matrix of shortest network path distances from each node to all other nodes in the network and repositions nodes so as to reduce the sum of the difference between the plotted distances and the network distances.[[3]](#endnote-4) A movie of the spread of flu with a frame for each of the 122 days of the study is available online at <http://jhfowler.ucsd.edu/flunet_v3.mov>.

While it is the case that, in situations of *chronic* illness, people that are sick may have fewer friends or different network architectures as a result of their illness, we do not anticipate a problem with this phenomenon in this setting. That is, we do not think that undergraduate friendships will be modified by virtue of having the flu, especially over the short time intervals being studied here.

**Personality Measures**

To measure self-perceived popularity, we adapted a set of 8 questions previously used to assess the popularity of co-workers.[[4]](#endnote-5) Specifically, we asked subjects to rate on a 5 point scale their agreement (ranging from *strongly disagree* to *strongly agree*) with the following statements: “I am popular,” “I am quite accepted,” “I am well-known,” “I am generally admired,” “I am liked,” “I am socially visible,” “I am viewed fondly,” and “I am not popular” (reverse scored). We generated index scores via a one-dimension factor analysis of all 8 items (Cronbach’s alpha=0.66).

**Analysis**

In Table S1 we report summary statistics for the random group and the friend group and the results of a Mann Whitney U test, which is a nonparametric test of differences in the two distributions. Notice that the friend group exhibits significantly higher in-degree and betweenness centrality, and significantly lower transitivity than the random group, as theorized. In addition, we find that the friend group has significantly more females and fewer sophomores than the random group.

In Table S2 we present Spearman correlations with *p* values to evaluate whether or not any study variables influence overall risk of getting the flu by December 31, 2009. Notice that the self-reported and medical staff measures are highly correlated at *ρ* = 0.40. However, no other variable is significantly associated with both measures. The two strongest associations with self-reported flu are in-degree and being a sophomore, but at 0.08 neither of these associations is strong and neither is confirmed in the data based on diagnoses by medical staff.

In Tables S3-S14, we report results from an estimation procedure designed to measure the shift in the time course of a contagious outbreak associated with a given independent variable. We fit the observed probability of flu to a cumulative logistic function

where *P_it_* is the probability subject *i* has the flu on or before day *t*; *t +α* + bX*_it_* is a function that determines the location of peak risk to subject *i* on day *t* that includes a constant *α*, a vector of coefficients b, and a matrix of independent variables X*_it_*; *σ* is a constant scale factor that provides an estimate of the standard deviation in days of the time course of the epidemic; and 0 ≤ *λ* ≤ 1 is a constant indicating the maximum cumulative risk. For medical diagnoses by staff, we assume *P_it_* is 1 when subjects have had the flu on any day up to and including *t* and 0 otherwise. For self-reported flu symptoms in some cases we only have information about the interval from *t_0_* to *t_1_* in which symptoms occurred, so we assume it increases uniformly in the interval, i.e. *P_it_* = ( *t* – *t_0_* ) / ( *t*_1_ – *t_0_* ).

To fit this equation we conducted a nonlinear least squares estimation procedure that utilizes the Gauss-Newton algorithm.[[5]](#endnote-6) To estimate standard errors and 95% confidence intervals, we used a bootstrapping procedure in which we repeatedly re-sampled subject observations with replacement and re-estimated the fit.[[6]](#endnote-7) This procedure produced somewhat wider confidence intervals than those derived from asymptotic approximations, so we report only the more conservative bootstrapped estimates of the standard errors in the Tables S3-S14.

In the left panel of Figure 2 we calculated the nonparametric maximum likelihood estimate (NPMLE) of cumulative flu incidence for both the friend group and the random group[[7]](#endnote-8) and in the right panel we show the predicted daily incidence based on Model 1 in Table S3. Daily incidence for the random group is the derivative of the cumulative logistic function:

and for the friends group is:

In Figure 4, we calculate early detection days for in-degree by multiplying the coefficient and confidence intervals in Table S7 by the difference in in-degree between the above-average-in-degree group and the below-average-in-degree group. Similarly, we calculate early detection days for betweenness by multiplying the coefficient and confidence intervals in Table S9 by the difference in betweenness between the above-average-betweenness group and the below-average-betweenness group. We calculate early detection days for *k-*coreness by multiplying the coefficient and confidence intervals in Table S11 by the difference in coreness between the above-average-coreness group and the below-average*-*coreness group. And we calculate early detection days for transitivity by multiplying the coefficient and confidence intervals in Table S13 by the difference in transitivity between the above-average-transitivity group and the below-average-transitivity group.

Table S1: Summary Statistics for Friend Group and Random Group

|  | *Friend Group* | | *Random Group* | | *Mann-Whitney U* |  |
| --- | --- | --- | --- | --- | --- | --- |
|  | *Mean* | *S.D.* | *Mean* | *S.D.* | *p* | *N* |
| Flu Diagnosis by Medical Staff | 0.075 | 0.264 | 0.078 | 0.269 | 0.876 | 744 |
| Self-Reported Flu Symptoms | 0.325 | 0.469 | 0.310 | 0.463 | 0.678 | 744 |
| In Degree | 1.435 | 0.663 | 0.433 | 0.664 | 0.000 | 744 |
| Out Degree | 2.689 | 0.543 | 2.611 | 0.672 | 0.306 | 744 |
| Betweenness Centrality (Percentile) | 0.559 | 0.271 | 0.423 | 0.294 | 0.000 | 744 |
| *K*-Coreness | 1.673 | 0.553 | 1.414 | 0.565 | 0.000 | 744 |
| Transitivity | 0.142 | 0.231 | 0.148 | 0.274 | 0.039 | 721 |
| Popularity Index | 4.053 | 0.982 | 3.967 | 1.022 | 0.195 | 744 |
| Self-Reported H1N1 Vaccine | 0.200 | 0.400 | 0.188 | 0.391 | 0.685 | 744 |
| H1N1 Vaccine at UHS | 0.115 | 0.320 | 0.110 | 0.313 | 0.812 | 744 |
| Self-Reported Seasonal Flu Vaccine | 0.499 | 0.528 | 0.473 | 0.506 | 0.595 | 744 |
| Seasonal Flu Vaccine at UHS | 0.388 | 0.488 | 0.401 | 0.491 | 0.719 | 744 |
| Female | 0.720 | 0.450 | 0.627 | 0.484 | 0.007 | 744 |
| Sophomore | 0.176 | 0.382 | 0.235 | 0.425 | 0.049 | 744 |
| Junior | 0.259 | 0.439 | 0.238 | 0.427 | 0.522 | 744 |
| Senior | 0.322 | 0.468 | 0.276 | 0.448 | 0.172 | 744 |
| Varsity Athlete | 0.092 | 0.289 | 0.113 | 0.317 | 0.345 | 744 |

Friend group *N*=425, random group *N*=325. The Mann Whitney U *p* value indicates the probability that values for the friends and random groups were drawn from the same distribution.

Table S2: Correlates of Getting Flu by December 31, 2009

|  | *Medical Staff Flu Diagnoses* | | *Self-Reported Flu Symptoms* | |
| --- | --- | --- | --- | --- |
|  | *Correlation* | *p* | *Correlation* | *p* |
| Flu Diagnosis by Medical Staff | --- | --- | 0.40 | 0.00 |
| Self-Reported Flu Symptoms | 0.40 | 0.00 | --- | --- |
| Member of Friend Group | -0.01 | 0.88 | 0.02 | 0.68 |
| In Degree | 0.01 | 0.78 | 0.08 | 0.02 |
| Out Degree | -0.01 | 0.75 | 0.01 | 0.84 |
| Betweenness Centrality | 0.02 | 0.67 | 0.03 | 0.36 |
| *K*-Coreness | 0.04 | 0.28 | 0.09 | 0.02 |
| Transitivity | -0.03 | 0.46 | 0.05 | 0.19 |
| Popularity Index | -0.03 | 0.46 | 0.01 | 0.86 |
| Self-Reported H1N1 Vaccine | -0.04 | 0.28 | -0.03 | 0.41 |
| H1N1 Vaccine at UHS | -0.01 | 0.85 | 0.05 | 0.19 |
| Self-Reported Seasonal Flu Vaccine | 0.01 | 0.75 | 0.04 | 0.33 |
| Seasonal Flu Vaccine at UHS | 0.05 | 0.20 | 0.05 | 0.16 |
| Female | 0.02 | 0.51 | 0.06 | 0.10 |
| Sophomore | 0.04 | 0.23 | 0.04 | 0.22 |
| Junior | -0.06 | 0.09 | 0.08 | 0.02 |
| Senior | -0.06 | 0.12 | -0.07 | 0.07 |
| Varsity Athlete | 0.04 | 0.30 | -0.04 | 0.31 |

*P* values indicate probability the Pearson correlation is 0. Lack of consistent correlation suggests none of the independent variables influence overall cumulative risk of flu.

Table S3: Effect of Being in the Friend Group on Cumulative Flu Incidence, Diagnoses by Medical Staff

|  | *Model 1* | | | | *Model 2* | | | |
| --- | --- | --- | --- | --- | --- | --- | --- | --- |
|  | *Coef.* | *S.E.* | *Lower 95% C.I.* | *Upper 95% C.I.* | *Coef.* | *S.E.* | *Lower 95% C.I.* | *Upper 95% C.I.* |
| *Location Variables:* |  |  |  |  |  |  |  |  |
| ***Friend Group*** | **-13.9** | **1.7** | **-16.6** | **-19.9** | **-15.7** | **2.1** | **-19.3** | **-12.4** |
| *H1N1 Vaccination* |  |  |  |  | 32.0 | 7.3 | 17.6 | 43.8 |
| *Seasonal Flu Vaccination* |  |  |  |  | -0.8 | 2.2 | -5.6 | 1.3 |
| *Female* |  |  |  |  | -8.5 | 2.2 | -12.2 | -3.2 |
| *Sophomore* |  |  |  |  | 25.2 | 2.9 | 20.1 | 32.1 |
| *Junior* |  |  |  |  | 66.7 | 3.1 | 61.2 | 72.8 |
| *Senior* |  |  |  |  | 57.6 | 2.5 | 54.3 | 62.3 |
| *Varsity Athlete* |  |  |  |  | -5.7 | 3.0 | -12.0 | -1.7 |
| *Constant* | 69.4 | 1.5 | 66.9 | 72.1 | 43.0 | 2.8 | 38.2 | 47.2 |
| *Scale Variable:* | 24.7 | 1.2 | 22.9 | 26.7 | 26.7 | 1.1 | 19.5 | 23.3 |
| *Residual Standard Error* | 0.2031 | | | | 0.2022 | | | |

Nonlinear least squares estimates of parameters in a cumulative logistic function fit to the cumulative incidence of flu diagnosed by medical staff in 744 subjects, each followed for 122 days. Location variable coefficients can be interpreted as the shift that occurs in days with respect to a unit increase in the independent variable. Standard errors and confidence intervals are bootstrapped. Results show friend group gets diagnosed with the flu by medical staff about 15 days earlier than the random group, and controlling for other factors does not affect the significance of the estimate.

Table S4: Effect of Being in the Friend Group on Cumulative Flu Incidence, Self-Reported Data

|  | *Model 3* | | | | *Model 4* | | | |
| --- | --- | --- | --- | --- | --- | --- | --- | --- |
|  | *Coef.* | *S.E.* | *Lower 95% C.I.* | *Upper 95% C.I.* | *Coef.* | *S.E.* | *Lower 95% C.I.* | *Upper 95% C.I.* |
| *Location Variables:* |  |  |  |  |  |  |  |  |
| *Friend Group* | -3.2 | 0.6 | -4.3 | -2.2 | -2.5 | 0.6 | -3.6 | -1.6 |
| *H1N1 Vaccination* |  |  |  |  | 12.5 | 1.2 | 10.1 | 14.8 |
| *Seasonal Flu Vaccination* |  |  |  |  | 2.8 | 0.5 | 1.8 | 3.9 |
| *Female* |  |  |  |  | -9.0 | 0.7 | -10.3 | -7.7 |
| *Sophomore* |  |  |  |  | -5.3 | 0.8 | -6.9 | -4.0 |
| *Junior* |  |  |  |  | -7.3 | 0.6 | -8.5 | -6.2 |
| *Senior* |  |  |  |  | 6.9 | 0.8 | 5.6 | 8.2 |
| *Varsity Athlete* |  |  |  |  | 6.6 | 0.8 | 5.1 | 8.2 |
| *Constant* | 123.9 | 0.6 | 122.9 | 125.2 | 126.2 | 1.0 | 124.1 | 128.6 |
| *Scale Variable:* | 36.8 | 0.4 | 36.1 | 37.4 | 34.9 | 0.3 | 34.4 | 35.5 |
| *Residual Standard Error* | 0.3481 | | | | 0.3463 | | | |

Nonlinear least squares estimates of parameters in a cumulative logistic function fit to the self-reported cumulative incidence of flu in 744 subjects, each followed for 122 days. Location variable coefficients can be interpreted as the shift that occurs in days with respect to a unit increase in the independent variable. Standard errors and confidence intervals are bootstrapped. Results show the friend group self-reports flu symptoms about 3 days earlier than the random group, and controlling for other factors does not affect the significance of the estimate.

Table S5: Effect of Self-Reported Popularity on Cumulative Flu Incidence

|  | *Model 5 (Medical Staff Diagnoses)* | | | | *Model 6 (Self Reports)* | | | |
| --- | --- | --- | --- | --- | --- | --- | --- | --- |
|  | *Coef.* | *S.E.* | *Lower 95% C.I.* | *Upper 95% C.I.* | *Coef.* | *S.E.* | *Lower 95% C.I.* | *Upper 95% C.I.* |
| *Location Variables:* |  |  |  |  |  |  |  |  |
| *Self-Reported Popularity* | 3.6 | 1.0 | 2.2 | 6.0 | -1.3 | 0.2 | -1.7 | -0.8 |
| *Constant* | 47.4 | 4.2 | 37.7 | 52.5 | 127.2 | 1.1 | 124.8 | 128.7 |
| *Scale Variable:* | 25.5 | 1.2 | 23.4 | 27.6 | 36.8 | 0.4 | 36.2 | 37.5 |
| *Residual Standard Error* | 0.2032 | | | | 0.3481 | | | |

Nonlinear least squares estimates of parameters in a cumulative logistic function fit to the cumulative incidence of flu diagnosed by medical staff (left model) and self-reported (right model) in 744 subjects, each followed for 122 days. Location variable coefficients can be interpreted as the shift that occurs in days with respect to a unit increase in the independent variable. Standard errors and confidence intervals are bootstrapped. Results self-reported popularity has an inconsistent effect on timing of the flu.

Table S6: Effect of Being in the Friend Group on Cumulative Flu Incidence, Controlling for Self-Reported Popularity

|  | *Model 7 (Medical Staff Diagnoses)* | | | | *Model 8 (Self Reports)* | | | |
| --- | --- | --- | --- | --- | --- | --- | --- | --- |
|  | *Coef.* | *S.E.* | *Lower 95% C.I.* | *Upper 95% C.I.* | *Coef.* | *S.E.* | *Lower 95% C.I.* | *Upper 95% C.I.* |
| *Location Variables:* |  |  |  |  |  |  |  |  |
| *Friend Group* | -14.5 | 2.4 | -19.4 | -11.0 | -3.1 | 0.5 | -4.1 | -2.2 |
| *Self-Reported Popularity* | 4.1 | 1.0 | 2.1 | 5.5 | -1.3 | 0.3 | -1.8 | -0.7 |
| *Constant* | 53.5 | 4.2 | 48.1 | 61.8 | 128.9 | 1.2 | 127.2 | 131.4 |
| *Scale Variable:* | 24.7 | 1.0 | 23.0 | 26.3 | 36.8 | 0.4 | 36.2 | 37.6 |
| *Residual Standard Error* | 0.2031 | | | | 0.3480 | | | |

Nonlinear least squares estimates of parameters in a cumulative logistic function fit to the cumulative incidence of flu diagnosed by medical staff (left model) and self-reported (right model) in 744 subjects, each followed for 122 days. Location variable coefficients can be interpreted as the shift that occurs in days with respect to a unit increase in the independent variable. Standard errors and confidence intervals are bootstrapped. Results show the friend group gets the flu significantly earlier, even when controlling for a self-reported measure of popularity.

Table S7: Effect of Network In Degree on Cumulative Flu Incidence

|  | *Model 9 (Medical Staff Diagnoses)* | | | | *Model 10 (Self Reports)* | | | |
| --- | --- | --- | --- | --- | --- | --- | --- | --- |
|  | *Coef.* | *S.E.* | *Lower 95% C.I.* | *Upper 95% C.I.* | *Coef.* | *S.E.* | *Lower 95% C.I.* | *Upper 95% C.I.* |
| *Location Variables:* |  |  |  |  |  |  |  |  |
| *In Degree* | -5.7 | 1.3 | -8.1 | -3.6 | -8.0 | 0.4 | -8.5 | -7.3 |
| *Constant* | 67.8 | 1.4 | 65.4 | 70.3 | 130.2 | 0.7 | 128.6 | 131.3 |
| *Scale Variable:* | 25.2 | 1.3 | 22.8 | 27.8 | 36.8 | 0.5 | 35.9 | 37.6 |
| *Residual Standard Error* | 0.2032 | | | | 0.3470 | | | |

Nonlinear least squares estimates of parameters in a cumulative logistic function fit to the cumulative incidence of flu diagnosed by medical staff (left model) and self-reported (right model) in 744 subjects, each followed for 122 days. Location variable coefficients can be interpreted as the shift that occurs in days with respect to a unit increase in the independent variable. Standard errors and confidence intervals are bootstrapped. Results show that individuals with high in-degree tend to get the flu earlier than others.

Table S8: Effect of Network Out Degree on Cumulative Flu Incidence

|  | *Model 11 (Medical Staff Diagnoses)* | | | | *Model 12 (Self Reports)* | | | |
| --- | --- | --- | --- | --- | --- | --- | --- | --- |
|  | *Coef.* | *S.E.* | *Lower 95% C.I.* | *Upper 95% C.I.* | *Coef.* | *S.E.* | *Lower 95% C.I.* | *Upper 95% C.I.* |
| *Location Variables:* |  |  |  |  |  |  |  |  |
| *Out Degree* | 7.5 | 1.8 | 4.7 | 11.2 | 2.5 | 0.4 | 1.5 | 3.2 |
| *Constant* | 42.2 | 4.8 | 32.9 | 49.5 | 115.2 | 1.3 | 113.3 | 118.3 |
| *Scale Variable:* | 25.5 | 1.2 | 23.0 | 27.4 | 36.8 | 0.4 | 36.0 | 37.4 |
| *Residual Standard Error* | 0.2032 | | | | 0.3481 | | | |

Nonlinear least squares estimates of parameters in a cumulative logistic function fit to the cumulative incidence of flu diagnosed by medical staff (left model) and self-reported (right model) in 744 subjects, each followed for 122 days. Location variable coefficients can be interpreted as the shift that occurs in days with respect to a unit increase in the independent variable. Standard errors and confidence intervals are bootstrapped. Results show that the number of friends a person nominates actually *delays* the average onset of flu.

Table S9: Effect of Betweenness Centrality on Cumulative Flu Incidence

|  | *Model 13 (Medical Staff Diagnoses)* | | | | *Model 14 (Self Reports)* | | | |
| --- | --- | --- | --- | --- | --- | --- | --- | --- |
|  | *Coef.* | *S.E.* | *Lower 95% C.I.* | *Upper 95% C.I.* | *Coef.* | *S.E.* | *Lower 95% C.I.* | *Upper 95% C.I.* |
| *Location Variables:* |  |  |  |  |  |  |  |  |
| *Betweenness Centrality* | -16.5 | 8.3 | -28.3 | -1.9 | -22.9 | 1.9 | -27.2 | -20.0 |
| *Constant* | 62.9 | 1.1 | 60.9 | 65.0 | 123.2 | 0.5 | 122.1 | 123.9 |
| *Scale Variable:* | 25.5 | 1.1 | 23.3 | 27.5 | 36.8 | 0.4 | 35.9 | 37.4 |
| *Residual Standard Error* | 0.2032 | | | | 0.3479 | | | |

Nonlinear least squares estimates of parameters in a cumulative logistic function fit to the cumulative incidence of flu diagnosed by medical staff (left model) and self-reported (right model) in 744 subjects, each followed for 122 days. Location variable coefficients can be interpreted as the shift that occurs in days with respect to a unit increase in the independent variable. Standard errors and confidence intervals are bootstrapped. Results show that individuals with high betweenness centrality tend to get the flu earlier than others.

Table S10: Effect of Betweenness Centrality on Cumulative Flu Incidence With Controls

|  | *Model 15 (Medical Staff Diagnoses)* | | | | *Model 16 (Self Reports)* | | | |
| --- | --- | --- | --- | --- | --- | --- | --- | --- |
|  | *Coef.* | *S.E.* | *Lower 95% C.I.* | *Upper 95% C.I.* | *Coef.* | *S.E.* | *Lower 95% C.I.* | *Upper 95% C.I.* |
| *Location Variables:* |  |  |  |  |  |  |  |  |
| *Betweenness Centrality* | -15.0 | 8.4 | -27.3 | -0.4 | -16.6 | 1.8 | -19.5 | -13.5 |
| *In Degree* | -4.2 | 1.3 | -6.2 | -1.4 | -7.6 | 0.4 | -8.3 | -6.9 |
| *Out Degree* | 7.8 | 1.7 | 5.4 | 11.4 | 3.4 | 0.5 | 2.6 | 4.3 |
| *Constant* | 46.7 | 4.7 | 37.0 | 54.0 | 121.6 | 1.3 | 118.9 | 124.3 |
| *Scale Variable:* | 25.3 | 1.2 | 23.2 | 27.5 | 36.7 | 0.4 | 35.9 | 37.4 |
| *Residual Standard Error* | 0.2031 | | | | 0.3468 | | | |

Nonlinear least squares estimates of parameters in a cumulative logistic function fit to the cumulative incidence of flu diagnosed by medical staff (left model) and self-reported (right model) in 744 subjects, each followed for 122 days. Location variable coefficients can be interpreted as the shift that occurs in days with respect to a unit increase in the independent variable. Standard errors and confidence intervals are bootstrapped. Results show that betweenness centrality remains a significant predictor of early flu onset even when controlling for degree variables.

Table S11: Effect of K-Coreness on Cumulative Flu Incidence

|  | *Model 13 (Medical Staff Diagnoses)* | | | | *Model 14 (Self Reports)* | | | |
| --- | --- | --- | --- | --- | --- | --- | --- | --- |
|  | *Coef.* | *S.E.* | *Lower 95% C.I.* | *Upper 95% C.I.* | *Coef.* | *S.E.* | *Lower 95% C.I.* | *Upper 95% C.I.* |
| *Location Variables:* |  |  |  |  |  |  |  |  |
| *K-Coreness* | -3.4 | 1.8 | -6.9 | -0.2 | -6.3 | 0.5 | -7.1 | -5.3 |
| *Constant* | 67.4 | 2.8 | 62.6 | 71.7 | 132.0 | 0.9 | 130.1 | 133.4 |
| *Scale Variable:* | 25.7 | 0.9 | 23.8 | 27.0 | 36.8 | 0.4 | 36.0 | 37.4 |
| *Residual Standard Error* | 0.2032 | | | | 0.3478 | | | |

Nonlinear least squares estimates of parameters in a cumulative logistic function fit to the cumulative incidence of flu diagnosed by medical staff (left model) and self-reported (right model) in 744 subjects, each followed for 122 days. Location variable coefficients can be interpreted as the shift that occurs in days with respect to a unit increase in the independent variable. Standard errors and confidence intervals are bootstrapped. Results show that individuals with high *k*-coreness tend to get the flu earlier than others.

Table S12: Effect of K-Coreness on Cumulative Flu Incidence With Controls

|  | *Model 15 (Medical Staff Diagnoses)* | | | | *Model 16 (Self Reports)* | | | |
| --- | --- | --- | --- | --- | --- | --- | --- | --- |
|  | *Coef.* | *S.E.* | *Lower 95% C.I.* | *Upper 95% C.I.* | *Coef.* | *S.E.* | *Lower 95% C.I.* | *Upper 95% C.I.* |
| *Location Variables:* |  |  |  |  |  |  |  |  |
| *K-Coreness* | -4.7 | 2.4 | -8.7 | -0.0 | -1.5 | 0.6 | -2.6 | -0.6 |
| *In Degree* | -4.3 | 1.4 | -6.5 | -1.8 | -7.5 | 0.4 | -8.2 | -6.8 |
| *Out Degree* | 8.5 | 1.9 | 4.5 | 11.4 | 3.2 | 0.5 | 2.4 | 4.1 |
| *Constant* | 51.6 | 5.1 | 43.1 | 62.5 | 123.5 | 1.4 | 121.3 | 125.8 |
| *Scale Variable:* | 25.4 | 1.2 | 23.3 | 28.3 | 36.7 | 0.4 | 35.9 | 37.4 |
| *Residual Standard Error* | 0.2031 | | | | 0.3469 | | | |

Nonlinear least squares estimates of parameters in a cumulative logistic function fit to the cumulative incidence of flu diagnosed by medical staff (left model) and self-reported (right model) in 744 subjects, each followed for 122 days. Location variable coefficients can be interpreted as the shift that occurs in days with respect to a unit increase in the independent variable. Standard errors and confidence intervals are bootstrapped. Results show that *k*-coreness remains a significant predictor of early flu onset even when controlling for degree variables.

Table S13: Effect of Transitivity on Cumulative Flu Incidence

|  | *Model 17 (Medical Staff Diagnoses)* | | | | *Model 18 (Self Reports)* | | | |
| --- | --- | --- | --- | --- | --- | --- | --- | --- |
|  | *Coef.* | *S.E.* | *Lower 95% C.I.* | *Upper 95% C.I.* | *Coef.* | *S.E.* | *Lower 95% C.I.* | *Upper 95% C.I.* |
| *Location Variables:* |  |  |  |  |  |  |  |  |
| *Transitivity* | 31.9 | 4.8 | 23.5 | 43.5 | 15.0 | 1.6 | 12.7 | 18.5 |
| *Constant* | 56.9 | 1.5 | 53.5 | 59.0 | 153.9 | 1.2 | 151.3 | 155.8 |
| *Scale Variable:* | 24.8 | 0.8 | 23.3 | 26.6 | 40.5 | 0.7 | 39.1 | 41.7 |
| *Residual Standard Error* | 0.2046 | | | | 0.2873 | | | |

Nonlinear least squares estimates of parameters in a cumulative logistic function fit to the cumulative incidence of flu diagnosed by medical staff (left model) and self-reported (right model) in 721 subjects, each followed for 122 days. Location variable coefficients can be interpreted as the shift that occurs in days with respect to a unit increase in the independent variable. Standard errors and confidence intervals are bootstrapped. Results show that individuals with low transitivity tend to get the flu earlier than others.

Table S14: Effect of Transitivity on Cumulative Flu Incidence with Controls

|  | *Model 19 (Medical Staff Diagnoses)* | | | | *Model 20 (Self Reports)* | | | |
| --- | --- | --- | --- | --- | --- | --- | --- | --- |
|  | *Coef.* | *S.E.* | *Lower 95% C.I.* | *Upper 95% C.I.* | *Coef.* | *S.E.* | *Lower 95% C.I.* | *Upper 95% C.I.* |
| *Location Variables:* |  |  |  |  |  |  |  |  |
| *Transitivity* | 34.9 | 4.2 | 25.1 | 42.0 | 22.8 | 1.8 | 19.1 | 25.6 |
| *In Degree* | -3.6 | 1.2 | -5.4 | -1.0 | -4.6 | 0.5 | -5.6 | -3.8 |
| *Out Degree* | 17.6 | 2.2 | 13.7 | 21.3 | 17.3 | 0.7 | 16.1 | 19.0 |
| *Constant* | 13.2 | 6.0 | 2.2 | 21.1 | 109.7 | 1.9 | 106.9 | 112.8 |
| *Scale Variable:* | 25.0 | 1.0 | 23.1 | 26.9 | 39.4 | 0.7 | 38.1 | 41.0 |
| *Residual Standard Error* | 0.2045 | | | | 0.2860 | | | |

Nonlinear least squares estimates of parameters in a cumulative logistic function fit to the cumulative incidence of flu diagnosed by medical staff (left model) and self-reported (right model) in 721 subjects, each followed for 122 days. Location variable coefficients can be interpreted as the shift that occurs in days with respect to a unit increase in the independent variable. Standard errors and confidence intervals are bootstrapped. Results show that transitivity remains a significant predictor of early flu onset even when controlling for degree variables.

**REFERENCES for SUPPORINTING INFORMATION**

1. . Freeman LC (1977) Set of measures of centrality based on betweenness. *Sociometry* 40:35-41. [↑](#endnote-ref-2)
2. . Batagelj V, Mrvar A (2006) *PAJEK: Program for Analysis and Visualization of Large Networks*, version 1.14 [↑](#endnote-ref-3)
3. . Kamada T, Kawai C (1989) An algorithm for drawing general undirected graphs. *Information Processing Letters* 31:113-120. [↑](#endnote-ref-4)
4. . Scott BA, Judge TA (2009) The popularity contest at work: who wins, why, and what do they receive? *J Applied Psych* 94: 20–33. [↑](#endnote-ref-5)
5. . Bates DM, Watts DG. *Nonlinear Regression Analysis and Its Applications* (New York: Wiley, 1988) [↑](#endnote-ref-6)
6. . Huet S, Bouvier A, Poursat MA, Jolivet E (2003) *Statistical tools for nonlinear regression: a practical guide with S-PLUS and R examples*. (Berlin: Springer). [↑](#endnote-ref-7)
7. . Turnbull BW (1976) The empirical distribution function with arbitrarily grouped, censored and truncated data. *J. R. Statist. Soc. B* 38: 290-295. [↑](#endnote-ref-8)
